# Training and external validation of pre-treatment FDG PET-CT-based models for outcome prediction in anal squamous cell carcinoma

**DOI:** 10.1007/s00330-023-10340-9

**Published:** 2023-11-04

**Authors:** Russell Frood, Joseph Mercer, Peter Brown, Ane Appelt, Hitesh Mistry, Rohit Kochhar, Andrew Scarsbrook

**Affiliations:** 1 Department of Radiology, Leeds Teaching Hospitals NHS Trust, Leeds, UK; 2 Leeds Institute of Medical Research at St James’s, University of Leeds, Leeds, UK; 3 Department of Radiology, The Christie NHS Foundation Trust, Manchester, UK; 4 Department of Radiology, York and Scarborough Teaching Hospitals NHS Foundation Trust, York, UK; 5 Division of Pharmacy, University of Manchester, Manchester, UK

**Keywords:** Squamous cell carcinoma, Anal canal, Positron emission tomography computed tomography, Event-free survival

## Abstract

**Objectives:**

The incidence of anal squamous cell carcinoma (ASCC) is increasing worldwide, with a significant proportion of patients treated with curative intent having recurrence. The ability to accurately predict progression-free survival (PFS) and overall survival (OS) would allow for development of personalised treatment strategies. The aim of the study was to train and external test radiomic/clinical feature derived time-to-event prediction models.

**Methods:**

Consecutive patients with ASCC treated with curative intent at two large tertiary referral centres with baseline FDG PET-CT were included. Radiomic feature extraction was performed using LIFEx software on the pre-treatment PET-CT. Two distinct predictive models for PFS and OS were trained and tuned at each of the centres, with the best performing models externally tested on the other centres’ patient cohort.

**Results:**

A total of 187 patients were included from centre 1 (mean age 61.6 ± 11.5 years, median follow up 30 months, PFS events = 57/187, OS events = 46/187) and 257 patients were included from centre 2 (mean age 62.6 ± 12.3 years, median follow up 35 months, PFS events = 70/257, OS events = 54/257). The best performing model for PFS and OS was achieved using a Cox regression model based on age and metabolic tumour volume (MTV) with a training c-index of 0.7 and an external testing c-index of 0.7 (standard error = 0.4).

**Conclusions:**

A combination of patient age and MTV has been demonstrated using external validation to have the potential to predict OS and PFS in ASCC patients.

**Clinical relevance statement:**

A Cox regression model using patients’ age and metabolic tumour volume showed good predictive potential for progression-free survival in external testing. The benefits of a previous radiomics model published by our group could not be confirmed on external testing.

**Key Points:**

*• A predictive model based on patient age and metabolic tumour volume showed potential to predict overall survival and progression-free survival and was validated on an external test cohort.*

*• The methodology used to create a predictive model from age and metabolic tumour volume was repeatable using external cohort data.*

*• The predictive ability of positron emission tomography-computed tomography–derived radiomic features diminished when the influence of metabolic tumour volume was accounted for.*

**Supplementary Information:**

The online version contains supplementary material available at 10.1007/s00330-023-10340-9.

## Introduction

Anal carcinoma, although rare, is increasing in incidence worldwide with the most common histological type being squamous cell carcinoma (ASCC) [1–3]. Since three landmark randomised trials between 1987 and 1994, the gold standard treatment for non-metastatic ASCC is chemoradiotherapy with only very early anal margin tumours being excised [4–7]. Following the adoption of chemoradiotherapy as the standard treatment, the rate of loco-regional failure (LRF) decreased and there was improvements in overall survival (OS) and cancer-specific survival. However, there are still approximately 16% of patients with LRF, and 5-year mortality remains 25% [8]. The ability to accurately predict outcome at diagnosis could guide more tailored treatment, help stratify surveillance plans and ultimately improve outcomes.

European Society for Medical Oncology (ESMO) guidelines recommend pre-treatment use of multi-parametric magnetic resonance imaging (MRI) and 2-deoxy-2-[fluorine-18]fluoro-d-glucose (FDG) positron emission tomography/computed tomography (PET-CT) to assess regional and distant disease [9, 10]. These images provide the opportunity to extract quantitative data which can be used as features within a predictive model, a process termed as radiomics [11]. Although use of radiomics is widely reported for assessment and prediction in many different disease processes, limited data is available on outcome prediction models in ASCC using radiomic features extracted from PET-CT [12]. Brown et al used an elastic net model, combining least absolute shrinkage and selection operator (LASSO) and ridge regression for selection of radiomic features to predict progression-free survival (PFS) in ASCC patients treated with radiotherapy, mitomycin C and 5-fluorouracil regimens [13]. The study was based on retrospective data from a large tertiary centre with 145 patients in the training dataset and 44 patients in the internal test set. The model achieved a training area under the curve (AUC), based on the receiver operator characteristic (ROC) curve, of 0.74 and a test AUC of 0.73. However, the model was not externally validated. This is often a limitation in the published literature and means that it is not possible to determine how generalisable or universally applicable the reported model is [14].

This study aimed to derive and externally validate a predictive model for OS and PFS in ASCC patients using data from two large tertiary centres within the UK, Leeds Teaching Hospitals NHS Trust (LTHT) and the Christie NHS Foundation Trust (CNFT).

## Material and methods

The transparent reporting of a multivariable prediction model for individual prognosis or diagnosis (TRIPOD) guidelines were adhered to as part of the study (Supplementary Material 1).

### Patient selection

Consecutive patients with histological proven ASCC who underwent pre-treatment FDG PET/CT at LTHT between June 2008 and January 2017, or at CNFT between January 2012 and January 2018, were included. This allowed sufficient follow-up time for events to present in this cohort of patients. Exclusion criteria included patients with no definable tracer uptake in the primary tumour; treatment prior to PET-CT; patients not treated with curative intent; patients with incomplete clinical datasets; or if the primary lesion was too small to accurately segment on imaging.

Patient age, gender, clinical history, treatment history, clinical outcome and follow-up duration were recorded from the electronic patient records. PFS was recorded as any relapse, recurrence, or death from any cause. OS was recorded as death from any cause.

### Ethical consideration

All patients included within the study gave prospective consent at the time of imaging for their anonymised FDG PET-CT imaging data to be used in research and service development projects. Formal ethics committee approval was waived for this study for LTHT patients, as it was considered by the institutional review board to represent evaluation of a routine clinical service. For CNFT patients, use of data was approved by the institutional UK Computer Aided Theragnostics (ukCAT) ethics committee.

### Radiomic feature analysis

The details of the imaging protocols and who performed the segmentation and interpretation of images is available in Supplementary Material 2.

### Image segmentation and resampling

**Fig. 1:**
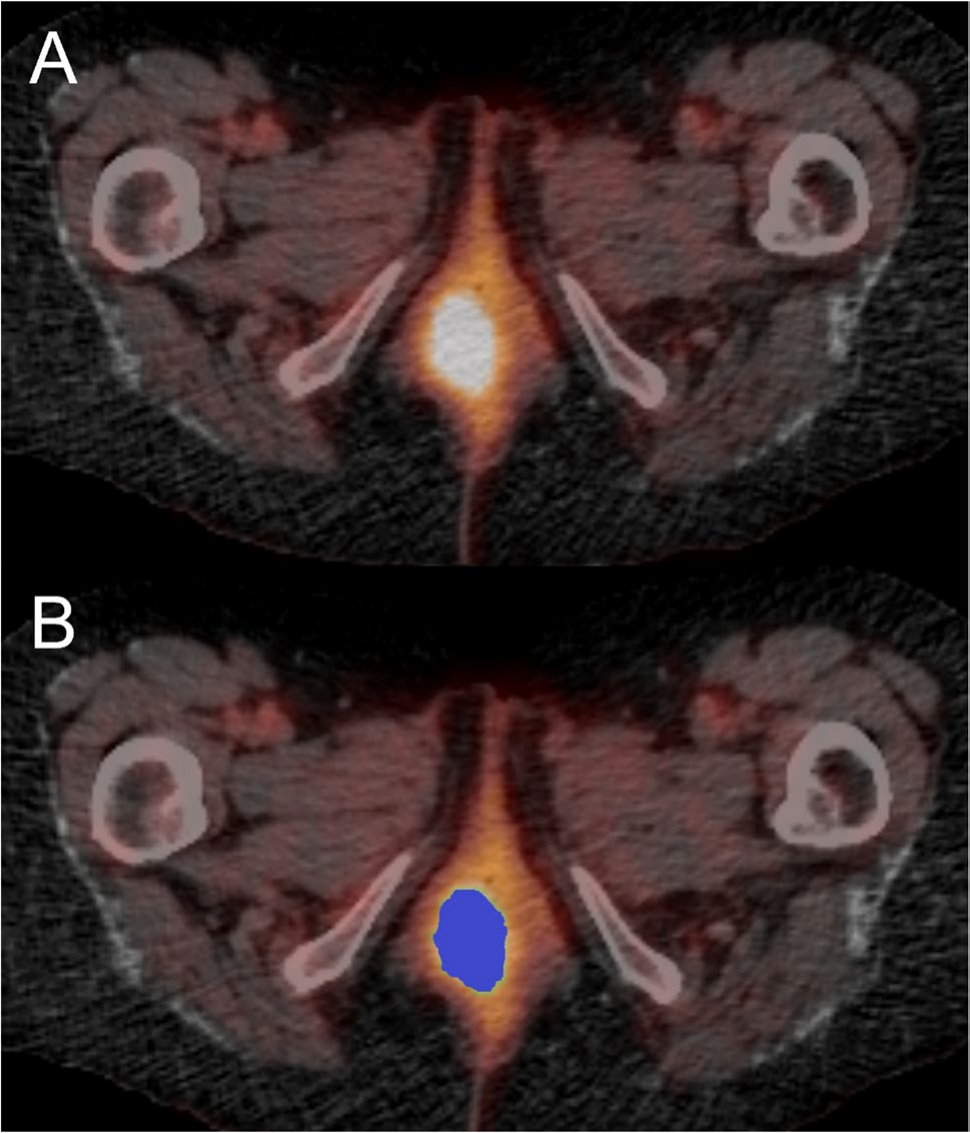
Axial PET-CT slices demonstrating a primary anal carcinoma mass (**A**) and the overlying mask created from a 1.5 times mean liver SUV (**B**)

Lesions were segmented with a semi-automated process using Local Image Features Extraction software (LIFEx v4.0, www.lifexsoft.org) [15]. There is no consensus on the optimal segmentation methodology in this clinical scenario. Whilst a fixed threshold may be easily applied, there is growing evidence that thresholding adapted to background physiological uptake might be more patient specific. In previous work, we have employed this segmentation methodology and have generally found that a threshold adapted to background physiological liver tracer uptake gives a good representation of lesions without background involvement which correlates to anatomical volumes on the CT component [13, 16, 17] (Fig. 1). Primary tumour region of interests ROI (t-ROI) and separate lymph-node ROI (ln-ROI) were contoured using a threshold of 1.5 × mean liver standardised uptake value (SUV) [18]. PET contours were transposed onto the co-registered CT to create CT ROIs. No lesions were excluded from analysis using this chosen segmentation threshold in the study cohort.

For each PET ROI, the voxel value was resampled into 64 bins ranging from 0 to 20 SUVs. The CT voxel values were resampled into 400 bins ranging from − 1000 to 3000 Hounsfield units (HU). For the LTHT dataset, the voxel dimensions were resampled to 4.0 × 4.0 × 4.0 mm for PET images and 2.5 × 1.2 × 1.2 mm (4.0 × 1.2 × 1.2 mm before June 2014) for CT images. For the CNFT dataset, the voxel dimensions were resampled to 4.0 × 4.0 × 4.0 mm for PET and CT images. Uniform resampling has been demonstrated to improve robustness in radiomic features when images are acquired using different voxel sizes [19].

### Feature extraction

Feature extraction was performed using LIFEx software which adheres to the image biomarker standardisation initiative (IBSI) [20]. Forty-four features were extracted for each PET and CT t-ROI, listed in Supplementary Material 2. Radiomic features were not extracted from the ln-ROI, but the volume of the ROI was used to calculate the total metabolic tumour volume (MTV).

### Statistical analysis and predictive modelling

Each model was trained, and hyperparameters tuned where applicable, initially on a single site, either LTHT or CNFT. Modelling and statistical analysis were performed using both R v4.0 and Python v3.7. A significant *p* value was taken as 0.05 except when Bonferroni correction was applied. Hazard ratios (HR) with 95% confidence intervals and c-indices with standard error were reported.

### CNFT predictive models

Firstly, a multivariable Cox proportional hazards (PH) regression model was created using clinical variables, tumour (T) stage, lymph node (N) stage, age and MTV using only complete cases. T and N-stage, age and MTV were considered continuous variables. The proportional hazards assumption was assessed graphically, and variables transformed if necessary. Secondly, unsupervised, and supervised learning methods were then used to explore the development of a radiomics-based Cox model. The unsupervised approach used principal component analysis (PCA) on the correlation matrix to reduce the dimensionality of the radiomics dataset. The leading principal components that accounted for 80% of the variability were explored using a Cox PH model with/without adjusting for MTV for both PFS and OS. The supervised learning approach involved exploring each radiomic feature in univariable analysis with/without adjusting for MTV. Any significant features were adjusted for the baseline clinical model for OS or PFS. For each final model, calibration plots comparing observed versus fitted survival probabilities over time were assessed.

### LTHT predictive models

A more automated pipeline with integrated harmonisation of the data for training and tuning of ML models for prediction of OS and PFS was explored. Models were created by dummy encoding the categorical clinical features (pandas.get_dummies v1.2.1), scaling the continuous features (sklearn.preprocessing.standardscaler v0.24.1) and harmonising the PET-CT radiomic features by applying neuroCombat to the extracted data (https://github.com/Jfortin1/ComBatHarmonization). To avoid redundancy, features with a Pearson coefficient of > 0.8 were removed. Three different ML models were evaluated on the dataset: survival support vector machine (SSVM), survival random forest (SRF) and Cox regression (scikit-survival v0.14.0).

A forward wrapper method was implemented for feature selection using threefold cross-validation (sklearn.model_selection.repeatedkfold v0.24.1). The optimum number of features for the greatest c-index for each ML algorithm was selected and hyperparameters were tuned using a grid search with a threefold cross-validation with 10 repeats (sklearn.model_selection.gridsearchcv v0.24.1).

### External testing

No data extracted from the images were shared between sites. The models with the highest c-index for each method created at a single institution were re-tested on external data at the other institution. Kaplan–Meier survival plots were produced from the external test-set.

### Repeatability of the methodology

The methodology for the best overall performing prediction model from either site was applied to the dataset of the other institution to determine if the same features would be selected and if the model would achieve a similar prediction score when trained on a different study population.

## Results

### Patient demographics

One hundred and eighty-seven patients were included from LTHT and 257 patients included from CNFT, with the median follow-up time being 30 months (IQR = 38 months) at LTHT and 45 months (IQR = 27 months) at CNFT. The breakdown of patient demographics is included in Table 1.

**Table 1:** Basic demographics of both study groups. The comparison between the two study groups was performed using a *t*-test for continuous data and a chi-square test for the categorical data. *OS* overall survival, *PFS* progression-free survival, *SD* standard deviation, *VMAT* volumetric modulated arc therapy

	LTHT	CNFT	*p* value
No. of patients	187	257	
Age (mean ± SD)	61.6 ± 11.5	62 ± 12.3	0.73
Sex			
Male	62	81	0.79
Female	125	176	
Tumour stage			
T1	8	18	
T2	78	114	
T3	58	49	0.005
T4	43	67	
Unknown	0	9	
Lymph node stage			
N0	88	128	
N1	36	58	
N2	42	35	0.09
N3	21	33	
`Unknown	0	3	
Metastatic stage			
M0	177	249	0.33
M1	10	8	
Treatment			
Chemoradiotherapy	187	244	
Radiotherapy alone	0	10	< 0.001
Sequential	0	3	
Radiotherapy type			
Parallel pair	146	224	0.01
VMAT	41	33	
Events			
OS	46	54	0.97
PFS	57	70	

### CNFT predictive models

#### Baseline clinical model

All features within the clinical model were significant predictors of OS and PFS following univariable analysis (Table 2). M stage was not included in the univariable analysis due to the low prevalence within the dataset (8/257). Treatment regime was not explored as a feature in the model due to the insufficient variance to provide meaningful information to the models being trained. Following multivariable analysis, it was found that only MTV and age remained significant predictors of OS and PFS. Furthermore, MTV correlated strongly with T, N-stage and MRI size (maximum single axis dimension recorded following MDT review). Therefore, the final clinical model consisted of just age and MTV (Table 3), which achieved a c-index of 0.70 and 0.68 for OS and PFS on the training data respectively.

**Table 2:** Univariable and multivariable OS and PFS analysis

Overall survival				
	Univariable		Multivariable	
	HR (95% CI)	*p* value	HR (95% CI)	*p* value
Age in years	1.03 (1.00–1.05)	0.034	1.05 (1.02–1.08)	0.003
T-stage				
4 v 3 v 2 v 1	1.85 (1.38–2.48)	< 0.001	1.05 (0.65–1.70)	0.829
N-stage				
3 v 2 v 1 v 0	1.38 (1.09–1.75)	0.007	1.12 (0.85–1.48)	0.432
MRI tumour size (maximum single axis dimension)	1.24 (1.10–1.39)	< 0.001	0.98 (0.76–1.28)	0.896
log(MTV)	1.72 (1.31–2.26)	< 0.001	1.49 (1.01–1.42)	0.007
Progression-free survival				
	HR (95% CI)	*p* value	HR (95% CI)	*p* value
Age in years	1.02 (1.00–1.04)	0.033	1.03 (1.01–1.06)	0.006
T-Stage				
4 v 3 v 2 v 1	1.97 (1.52–2.55)	< 0.001	1.06 (0.70–1.63)	0.775
N-stage				
3 v 2 v 1 v 0	1.34 (1.09–1.64)	0.006	1.14 (0.89–1.47)	0.301
MRI tumour size (maximal single axis dimension)	1.23 (1.12–1.36)	< 0.001	1.03 (0.82–1.47)	0.775
log(MTV)	1.70 (1.33–2.16)	< 0.001	1.69 (1.08–2.64)	0.021

**Table 3:** Final clinical OS and PFS models

	OS: c-index = 0.70		PFS: c-index = 0.68	
	HR (95% CI)	*p* value	HR (95% CI)	
Age in years	1.04 (1.01–1.06)	0.004	1.03 (1.01–1.05)	0.012
log(MTV)	1.78 (1.32–2.41)	< 0.001	1.74 (1.34–2.26)	< 0.001

#### Unsupervised radiomics model

The first 5 principal components accounted for 82% of the variability. The correlation of the leading components is seen in Table 4 and shows that once adjusted for MTV, none of the leading components were associated with OS or PFS. Therefore, this model was not explored any further within the analysis.

**Table 4:** Unadjusted and MTV adjusted OS and PFS HRs and *p* values for leading principal components (PC)

Overall survival (*N* = 208)				
	HR (95% CI)	*p* value	MTV Adj. HR (95% CI)	*p* value
PC1	2.01 (1.16–3.49)	0.013	1.02 (0.59–1.78)	0.939
PC2	2.71 (1.53–4.80)	< 0.001	1.01 (0.43–2.37)	0.978
PC3	2.33 (1.40–3.87)	0.001	0.85 (0.36–1.97)	0.700
PC4	2.49 (1.10–5.68)	0.029	1.35 (0.46–3.99)	0.583
PC5	1.12 (0.67–1.87)	0.666	0.63 (0.31–1.32)	0.223
Progression-free survival (*N* = 208)				
	HR (95% CI)	*p* value	MTV Adj. HR (95% CI)	*p* value
PC1	1.97 (1.22–3.18)	0.006	1.06 (0.64–1.75)	0.823
PC2	2.72 (1.63–4.52)	< 0.001	1.16 (0.52–2.56)	0.720
PC3	2.25 (1.43–3.55)	< 0.001	0.85 (0.40–1.82)	0.678
PC4	2.04 (0.99–4.20)	0.053	1.04 (0.39–2.77)	0.938
PC5	1.20 (0.77–1.85)	0.422	0.74 (0.40–1.37)	0.340

#### Supervised radiomics model

**Fig. 2:**
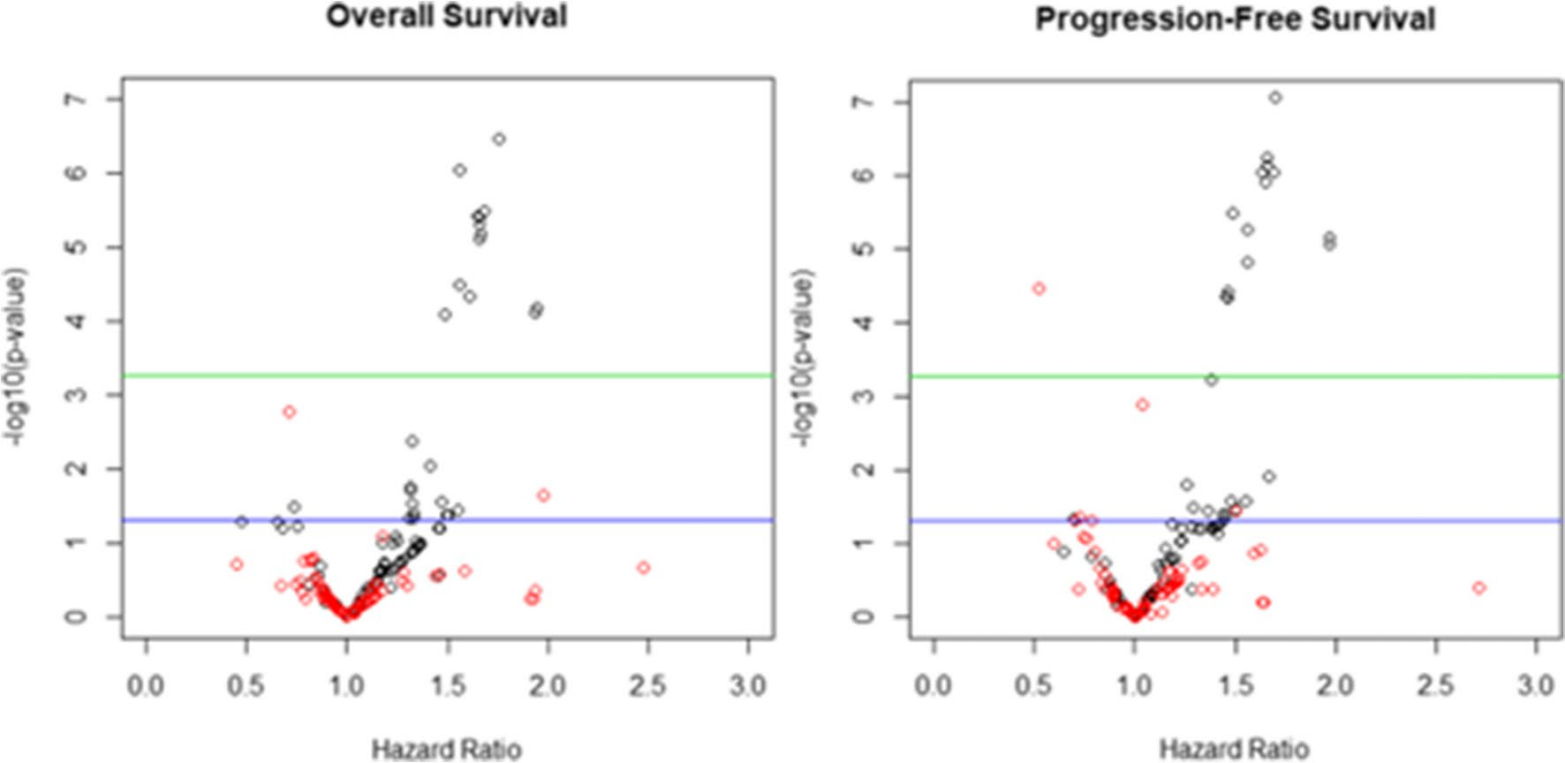
Volcano plots showing the relationship between *p* value and HR with (red circles) and without (black circles) adjusting for MTV. The blue line is the *p* value of 0.05 and the green line the Bonferroni corrected *p* value of 0.05

Each individual radiomic variable was individually explored in univariable analysis before and after adjusting for MTV. These results are visualised in the volcano plot in Fig. 2. Allowing for multiple testing (Bonferroni adjusted *p* value of 0.05, green line on plots below) but before adjustment for MTV, many radiomic features significantly correlate with both OS and PFS (black circles). However, the majority no longer do so once adjusted for MTV (red circles). Of interest, selected lower order PET features, total lesion glycolysis (TLG), SUV standard deviation and SUV_peak_ were close to the Bonferroni threshold for OS (TLG) and PFS (SUV standard deviation and SUV_peak_), respectively.

Upon adjustment for the clinical OS and PFS models, only TLG gave a modest improvement over the clinical model with no feature improving the PFS model. Therefore, a multivariable Cox regression model including age, MTV and TLG was created (Table 5) which achieved a c-index of 0.70 on the training dataset.

**Table 5:** Final radiomics OS model. *TLG was standardised—mean value 654 and standard deviation was 1193

	OS: c-index = 0.70	
	HR (95% CI)	*p* value
Age	1.03 (1.01–1.06)	0.019
log(MTV)	1.97 (1.44–2.71)	< 0.001
TLG*	0.80 (0.63–1.02)	0.072

#### External testing

**Fig. 3:**
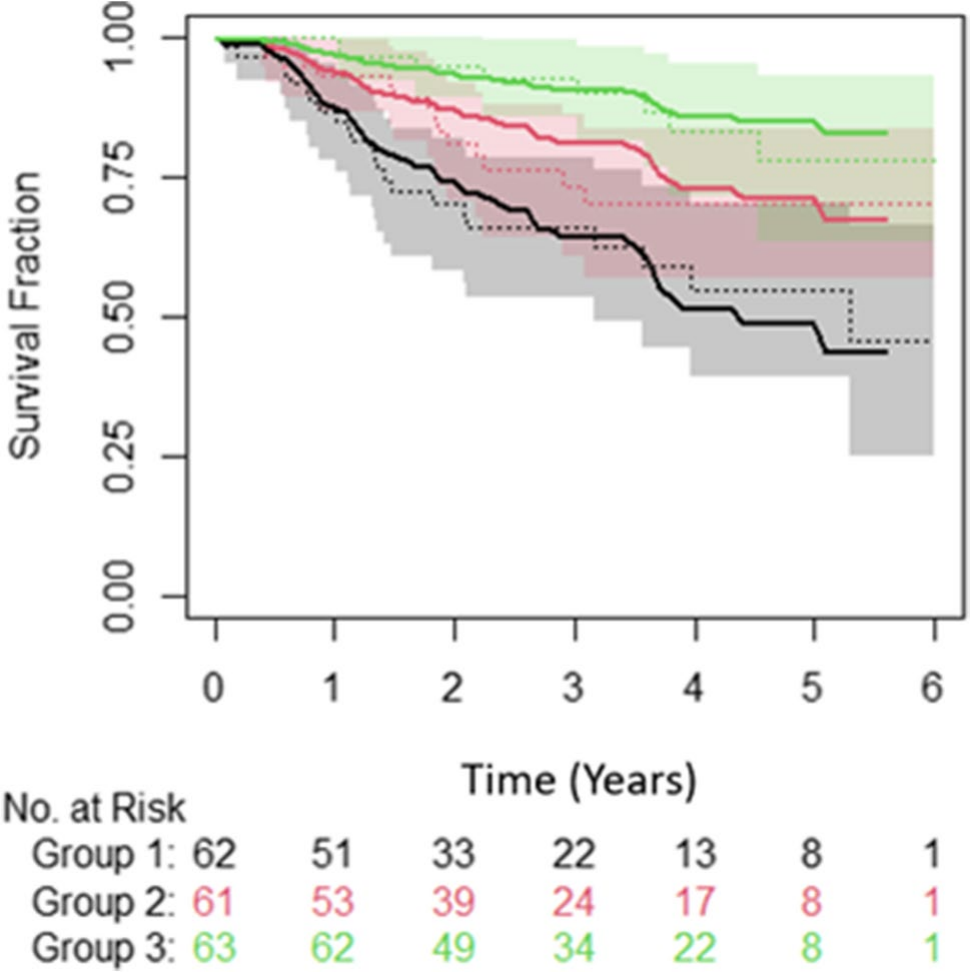
Plot showing the observed overall survival probabilities over time (dotted lines) with 95% confidence intervals (shaded region) and the predicted survival probabilities (solid lines) for a model created using MTV and age. Green = low-risk group, red = medium-risk group and black = high-risk group. Figure derived from the results of the testing on the LTHT dataset

Both the clinical and combined clinical- and radiomic-based OS models achieved a c-index of 0.70 on the training data and therefore were both tested on the LTHT dataset, with the clinical based model achieving a slightly higher c-index of 0.70 (S.E. = 0.4) compared to 0.69 (S.E. = 0.4) for the combined model. For both these models, the predictions of the low- and high-risk groups follow the actual event rates; however, the medium-risk group was either overestimated, age and MTV model, or underestimated, age, MTV and TLG. Also, the confidence intervals overlapped between all groups for both models, meaning no distinct groups could be defined (Fig. 3).

**Fig. 4:**
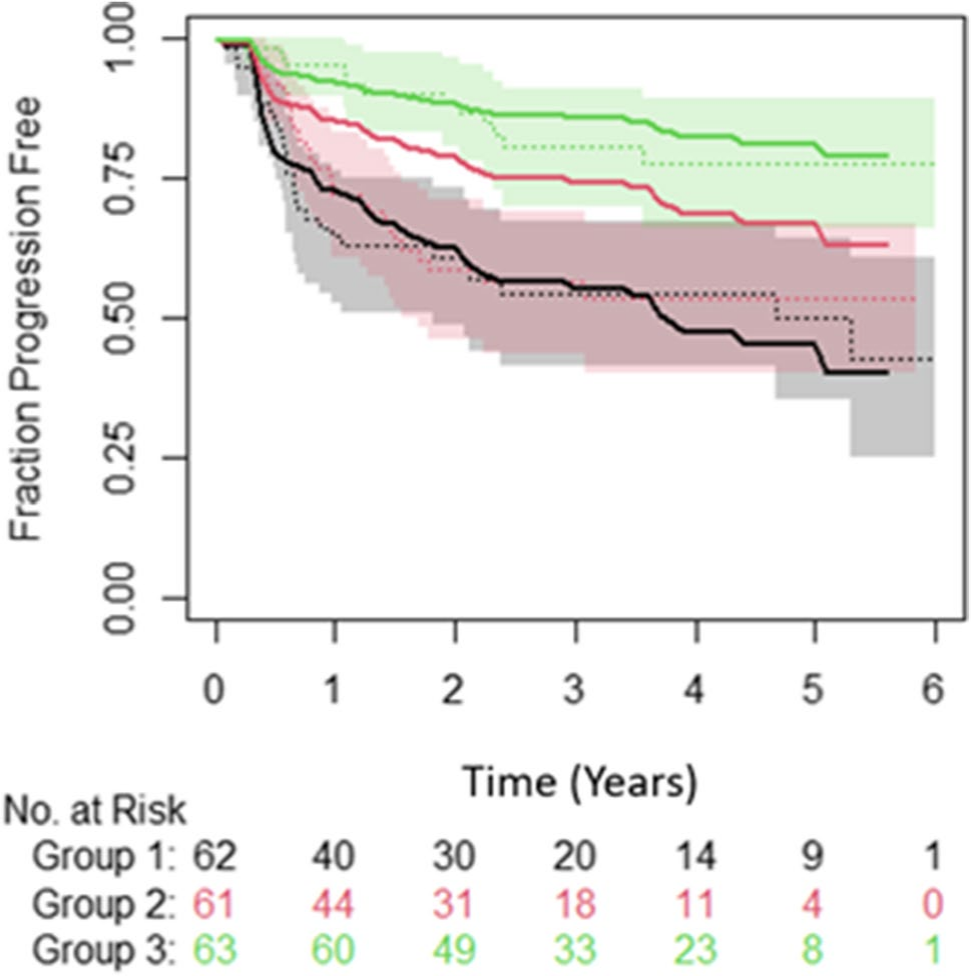
Plot showing the observed PFS over time (dotted lines) with 95% confidence intervals (shaded region) and the predicted (solid lines) for a model created using MTV and age. Green = low-risk group, red = medium-risk group and black = high-risk group. Figure derived from the results of testing on the LTHT dataset

When predicting PFS using a model derived from MTV and age, the c-index was 0.70 (S.E. = 0.4) on the test data with the model greatly overestimating the number of events of the medium-risk group (Fig. 4).

### LTHT predictive models

When using a forward wrapper method and ComBat harmonisation to explore three ML methods on the LTHT data, it was found that the best predictive model for OS was created using a SRF model with the parameters CT-derived neighbourhood grey-level difference matric (NGLDM) coarseness, age, CT-derived grey-level run length matrix (GLRLM), high grey run emphasis (HGRE), PET-derived grey-level co-occurrence matrix (GLCM) entropy log10 and CT-derived GLRLM short-run high grey-level emphasis (SRHGE). The hyperparameters used were random state = 0, bootstrap = True, maximum depth = 2, minimum number of samples per split = 4, maximum features = 5, minimum samples per leaf = 2, number of estimators = 50 and out of bag score = False. This led to a mean training c-index of 0.74.

No combination of features, ML model or hyperparameter selection allowed for the creation of a PFS model with a c-index above 0.55 without demonstrating obvious signs of overfitting (mean training and validation scores having a difference of over 0.20) and therefore no PFS model was tested on the external dataset.

#### External testing

The radiomic-based SRF OS prediction model had a test c-index of 0.60 when applied to the CNFT dataset. No PFS model was tested on the external dataset due to overfitting on the training dataset.

### Repeatability of the methodology

**Fig. 5:**
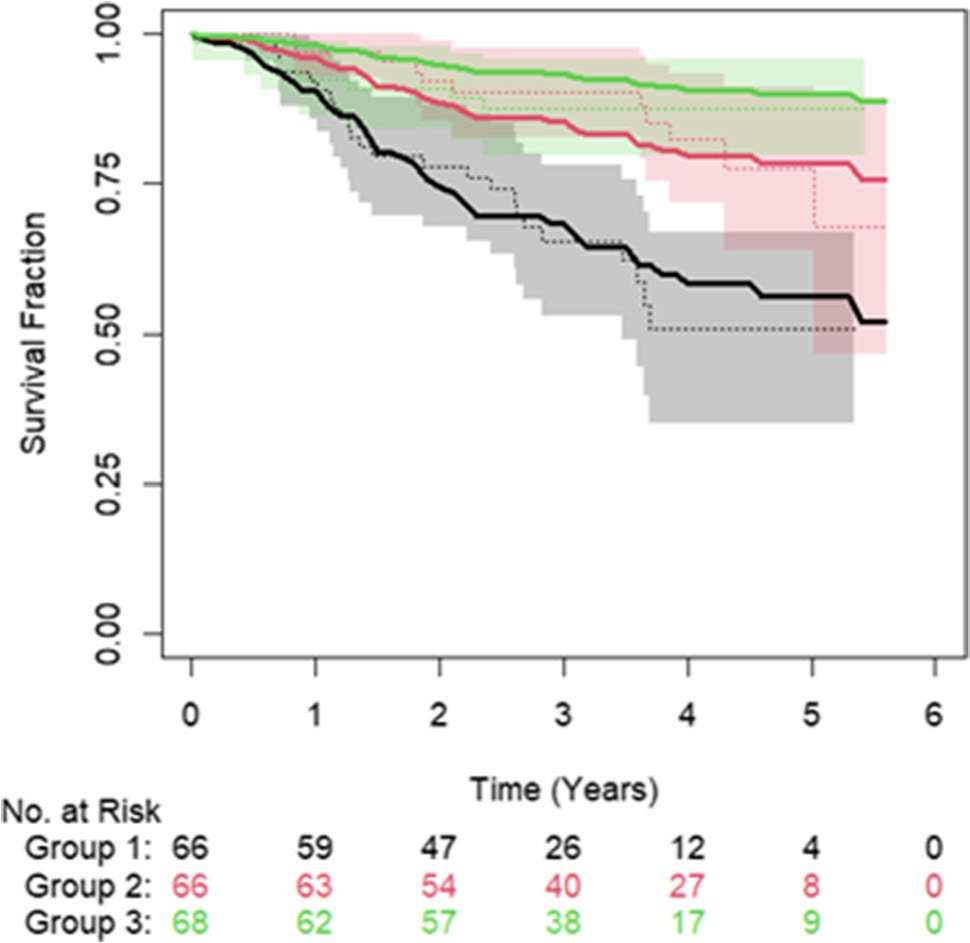
Plot showing the observed overall survival probabilities over time (dotted lines) with 95% confidence intervals (shaded region) and the predicted (solid lines) for a model created using MTV and age. Green = low-risk group, red = medium-risk group and black = high-risk group. Figure derived from the results of testing on the CNFT dataset

**Fig. 6:**
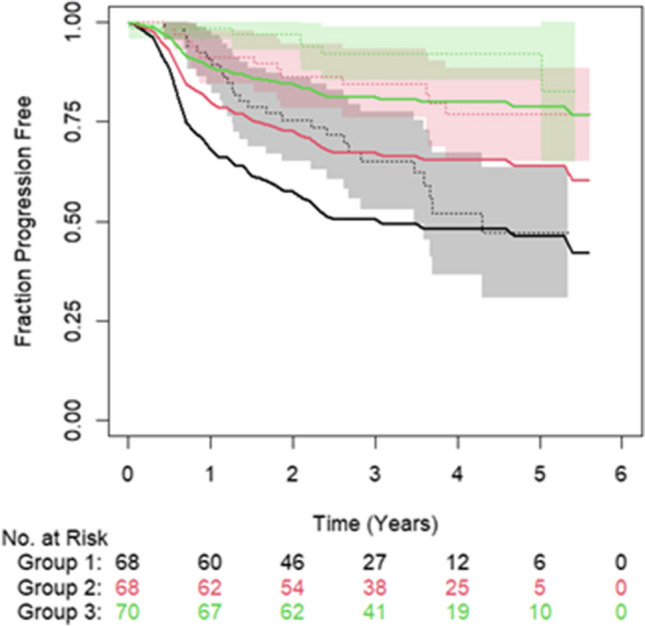
Plot showing the observed fraction progression free over time (dotted lines) with 95% confidence intervals (shaded region) and the predicted (solid lines) for a model created using MTV and age. Green = low-risk group, red = medium-risk group and black = high-risk group. Figure derived from the results of testing on the CNFT dataset

When applying the best performing CFNT methodology to train a model on the LTHT dataset, the same parameters MTV and age were selected from the univariable and multivariable analysis. The models produced a test c-index of 0.7 (S.E. = 0.04) for both PFS and OS when tested on the CNFT test set (Figs. 5 and 6). The predicted and observed survival probabilities were in good agreement for OS but not for PFS.

When investigating MTV and its relationship with outcomes, it was demonstrated that both cohorts had a non-linear relationship and therefore no definitive cutoff could be recommended. Representative plots are provided in Supplementary Material 3.

## Discussion

This study highlights MTV and age as biomarkers for predicting outcome in patients with ASCC, with MTV having a 1.49 HR and age having a 1.05 HR on multivariate analysis for OS and 1.69 HR and 1.03 HR for PFS, respectively. When combined in a Cox regression model, they had a good predictive ability with a c-index of 0.7 for both OS and PFS. Our study also illustrates the limitations of more complex radiomic based models when applying them to different populations.

The potential use of MTV for outcome prediction in this setting was first reported by Bazan et al in a small dataset of 39 patients [21]. Subsequent studies have explored the use of PET-CT-derived parameters in predictive models; however, due to the relatively low incidence of ASCC, study cohort sizes and event rates have been small. Of note, Rusten et al found that MTV, TLG and the Z-normalised combination of MTV and SUV_peak_ were predictive of outcomes, but the strongest predictors were nodal stage 3 disease and human papilloma virus (HPV) status [22]. Unfortunately, due to missing data within our cohort, it was not possible to explore HPV status further. Jones et al, in a prospective study, also reported that the predictive ability of MTV derived using a 41% SUVmax fixed threshold had excellent predictive ability for recurrence with an AUC of 0.89; however, this study only included 19 patients having a follow-up PET-CT 12 weeks after treatment [23]. Conversely, a larger study by Braun et al assessing outcomes in 60 patients found that MTV when split into two groups around the mean correlated well with T stage; however, there was no significant difference between disease-free survival [24].

There are various semi-automated segmentation techniques reported in the literature based on using a fixed SUV threshold, an adaptive threshold related to lesions being contoured or related to background physiological uptake. As far as we are aware, there is no published work comparing different segmentation approaches in anal cancer. Recent work defining the optimal method for MTV assessment of cervical cancer from pre-treatment FDG PET-CT evaluated different fixed and gradient segmentation methods showing excellent inter-observer agreement across all thresholds [25].

Our prior study exploring PET-CT-derived predictive modelling using radiomics in ASCC patients used the same 189 patient cohort as further analysed here [13]. We found that a regression-based model created from a combination of 10 different clinical and radiomic parameters, including MTV, had an AUC of 0.74 for the training and validation cohorts when predicting PFS. However, in the current study, we were not able to replicate this performance level, likely due to the presence of overfitting in the initial study, which was not identified as the model was trained and tested once on an internal dataset with a relatively small number of events. The issue of overfitting is highlighted in the current study whereby creation of models using a forward wrapper feature selection method in combination with a ML algorithm mean training and validation c-indices were > 0.7; however, when tested on external data, the c-index dropped to 0.6 at best. This is likely a combination of the limited number of events and the use of a relatively large number of parameters. To improve the model, a larger dataset with more events is required, which may allow the number of features used to create the model to be limited and a greater number of repeats of the cross validation could be performed to give a better estimation of how the model is performing [26]. This also illustrates the need for an external test set, as highlighted within the radiomics quality score, when evaluating predictive models as it is not possible to determine how generalisable the models are without these even with the use of cross-validation to assess stability [27]. However, it is noted that there is a relatively small number of ML studies which have published external testing [28, 29].

An MTV and age-based predictive model represents a simpler model which may be more generalisable than a complex model incorporating radiomic features. This could be a more pragmatic route to clinical translation but requires further validation in a multi-centre study. This aligns well with work being undertaken by the Anal cancer Treatment Outcome Modelling with Computer Aided Theragnositics (AtomCAT) consortium who have developed the infrastructure to support testing and validation of a clinical prediction model in anal cancer [30]. This presents an opportunity to test the applicability of a combined clinical and MTV-based model across a wide range of treatment centres. If proven to be more universally applicable, the model could have a significant impact on patient outcomes by guiding risk-adapted therapy and more personalised follow-up.

In our study, it was found that the predictive power of radiomics was lost when correcting for MTV and that the variation of the radiomic features was largely explained by MTV. This relationship was concordant across both sites data. However, this does not mean radiomic analysis should be discounted from future studies. The study limited itself to 44 features extracted from both the PET and CT components and used a fixed bin number of 64 bins and 400 bins when creating the matrix derived parameters for PET and CT, respectively. Numerous additional radiomic features have not been explored. There is no consensus on the optimum bin number or bin width, especially in ASCC; therefore, it may be prudent in future studies to look at the robustness of radiomic features in this clinical setting when using different bin widths [31, 32]. Also, when performing the initial univariable and multivariable analysis to assess the relationship of the features with outcome, only monotonic relationships were explored. Although the use of feature selection with models such as kernel version of the SSVM and SRF should negate this, the relationships between features and outcomes could be explored further.

Interpretation of the clinical significance of a prediction model can be challenging, and net benefit analysis can be used to determine clinical value [33]. This incorporates disease prevalence and weighting for consequences of false positive or false negative results and may provide a more representative measure of the clinical utility of including or excluding a test. Plotting net benefit over a range of appropriate weights to derive a decision curve could then be performed. This aspect was beyond the scope of the current work and is a study limitation.

Another consideration is the choice of outcome metrics, as the specific time that an event is recorded/diagnosed can vary greatly from when the patient developed recurrence or relapsed [34]. The choice of PFS and OS as outcome measures aligns with survival metrics being evaluated as part of the Core Outcome Research Measures in Anal Cancer (CORMAC) initiative and multi-centre prospective anal carcinoma radiotherapy trials being carried out at present [35, 36]. In prospective studies, this can be partially negated by having defined regular follow-up for all patients; however, this is not possible in retrospective series. The use of a binary cutoff value, for example 5-year PFS, would allow for a window for an event to occur meaning the need for accurate dating of recurrence or relapse is not as vital. However, this would limit the number of patients who met the minimum follow-up time and therefore limit the number of events; for this reason, a time-censored outcome was used in this study. In terms of OS, the time to death is more reliable and therefore could explain why in general the models for OS performed better, but it is based on all-cause mortality, and it could be argued some causes or mortality cannot be predicted on PET-CT.

## Conclusion

This study has demonstrated with external testing that a combination of age and MTV show potential in predicting OS and PFS in ASCC patients. The predictive ability of PET-CT-derived radiomic features diminished when the influence of MTV was accounted for.

### Supplementary Information

Below is the link to the electronic supplementary material.

Supplementary file1 (PDF 242 KB)

## Abbreviations

ASCC: Anal squamous cell carcinoma
AtomCAT: Anal cancer Treatment Outcome Modelling with Computer Aided Theragnostics
CNFT: Christie NHS Foundation Trust
CORMAC: Core Outcome Research Measures in Anal Cancer
ESMO: European Society for Medical Oncology
FDG: 2-Deoxy-2-[fluorine-18]fluoro-d-glucose
GLCM: Grey level co-occurrence matrix
GLRLM: Grey level run length matrix
GLSZM: Grey level size zone matrix
HPV: Human papilloma virus
HR: Hazard ratios
ln-ROI: Lymph-node ROI
LRF: Loco-regional failure
L-SUV_mean_: Mean liver standardised uptake value
LTHT: Leeds Teaching Hospitals NHS Trust
MTV: Metabolic tumour volume
NGTDM: Neighbouring grey tone difference matrix
OS: Overall survival
PCA: Principal component analysis
PET-CT: Positron emission tomography/computed tomography
PFS: Progression-free survival
PH: Proportional hazards
ROC: Receiver operator characteristic
ROI: Region of interest
SRF: Survival Random Forest
SSVM: Survival Support Vector Machine
t-ROI: Primary tumour ROI
ukCAT: UK Computer Aided Theragnostics

## Declarations

### Guarantor

The scientific guarantor of this publication is Prof A Scarsbrook.

### Statistics and biometry

One of the authors (Dr H Mistry) has significant statistical expertise.

### Study subjects or cohorts overlap

Some study subjects or cohorts have been previously reported. The dataset (187 patients) from Leeds has been used for a radiomics-based prediction model which has been published previously—Brown PJ, Zhong J, Frood R, et al (2019) Prediction of outcome in anal squamous cell carcinoma using radiomic feature analysis of pre-treatment FDG PET-CT. Eur J Nucl Med Mol Imaging 46: 2790–2799. However, this work looks at a range of different prediction models using the same data set representing new original research. In addition, the current study includes external validation with a large independent dataset not previously published.

### Methodology

• retrospective

• diagnostic or prognostic study

• multicenter study
