# Supplementary material for: Training and external validation of pre-treatment FDG PET-CT-based models for outcome prediction in anal squamous cell carcinoma
Source: Eur Radiol. 2023 Nov 4;34(5):3194–204. doi: 10.1007/s00330-023-10340-9 (PMC11126458; doi:10.1007/s00330-023-10340-9)
Supplement: Supplementary file 1 — Supplementary file1 (PDF 242 KB) [file 330_2023_10340_MOESM1_ESM.pdf]

Section/Topic		Checklist Item	Page
**Title and abstract**			
Title	1	Identify the study as developing and/or validating a multivariable prediction model, the target population, and the outcome to be predicted.	1
Abstract	2	Provide a summary of objectives, study design, setting, participants, sample size, predictors, outcome, statistical analysis, results, and conclusions.	4
**Introduction**			
Background and objectives	3a	Explain the medical context (including whether diagnostic or prognostic) and rationale for developing or validating the multivariable prediction model, including references to existing models.	5
	3b	Specify the objectives, including whether the study describes the development or validation of the model or both.	5
**Methods**			
Source of data	4a	Describe the study design or source of data (e.g., randomized trial, cohort, or registry data), separately for the development and validation data sets, if applicable.	6
	4b	Specify the key study dates, including start of accrual; end of accrual; and, if applicable, end of follow-up.	6
Participants	5a	Specify key elements of the study setting (e.g., primary care, secondary care, general population) including number and location of centres.	6
	5b	Describe eligibility criteria for participants.	6
	5c	Give details of treatments received, if relevant.	6
Outcome	6a	Clearly define the outcome that is predicted by the prediction model, including how and when assessed.	6
	6b	Report any actions to blind assessment of the outcome to be predicted.	*
Predictors	7a	Clearly define all predictors used in developing or validating the multivariable prediction model, including how and when they were measured.	8
	7b	Report any actions to blind assessment of predictors for the outcome and other predictors.	*
Sample size	8	Explain how the study size was arrived at.	8
Missing data	9	Describe how missing data were handled (e.g., complete-case analysis, single imputation, multiple imputation) with details of any imputation method.	6
Statistical analysis methods	10a	Describe how predictors were handled in the analyses.	8,9,10
	10b	Specify type of model, all model-building procedures (including any predictor selection), and method for internal validation.	8, 9, 10
	10d	Specify all measures used to assess model performance and, if relevant, to compare multiple models.	10
Risk groups	11	Provide details on how risk groups were created, if done.	8,9,10
**Results**			
Participants	13a	Describe the flow of participants through the study, including the number of participants with and without the outcome and, if applicable, a summary of the follow-up time. A diagram may be helpful.	6
	13b	Describe the characteristics of the participants (basic demographics, clinical features, available predictors), including the number of participants with missing data for predictors and outcome.	Tab 3
Model development	14a	Specify the number of participants and outcome events in each analysis.	Tab 3
	14b	If done, report the unadjusted association between each candidate predictor and outcome.	N/A
Model specification	15a	Present the full prediction model to allow predictions for individuals (i.e., all regression coefficients, and model intercept or baseline survival at a given time point).	11-18
	15b	Explain how to the use the prediction model.	11-18
Model performance	16	Report performance measures (with CIs) for the prediction model.	11-18
**Discussion**			
Limitations	18	Discuss any limitations of the study (such as nonrepresentative sample, few events per predictor, missing data).	18-20
Interpretation	19b	Give an overall interpretation of the results, considering objectives, limitations, and results from similar studies, and other relevant evidence.	18-20
Implications	20	Discuss the potential clinical use of the model and implications for future research.	18-20
**Other information**			
Supplementary information	21	Provide information about the availability of supplementary resources, such as study protocol, Web calculator, and data sets.	
Funding	22	Give the source of funding and the role of the funders for the present study.	N/A

This study looks at the training and external validation of pre-treatment PET-CT based model for outcome prediction anal squamous cell carcinoma. (**Title**)

The abstract covers a summary of all the requested information. (**Abstract**)

a) The introduction presents the background of squamous cell carcinoma (ASCC), although a rare cancer it has a low 5-year survival rate. (**Introduction**)
b) The aim of this study was to create and externally validate a predictive model using radiomic features derived from pre-treatment FDG PET/CT to predict EFS and OS in ASCC patients using data from two tertiary centre cohort of patients (**Introduction**)

a) This is a retrospective multi centre cohort study. Each centre providing the training, and then the external validation datasets for the other. (**Patient selection**)

b) Consecutive patients with histologically proven ASCC who underwent baseline FDG-PET/CT at LTHT between June 2008 and January 2017 were include or at CNFT between January 2012 and January 2018. The follow up information recorded is set out in the patient selection section. (**Patient selection**)

a) This is a multi-centre study. (**Patient selection**)
b) Exclusion criteria included patients with no definable tracer uptake in the primary tumour; treatment prior to the PET-CT; patients not treated with curative intent; or if the primary lesion was too small to accurately segment on imaging. (**Patient selection**)
c) No change to departmental standard treatment was performed.

a) PFS was recorded as any relapse, recurrence or death from any cause. OS was recorded as death from any cause. (**Patient selection**)
b) As this was a retrospective study the primary outcomes were defined from clinical records. The investigator reviewing the records was blinded to the imaging parameters.

a) The description of the contouring method, resampling, harmonisation, radiomic feature extraction and the methods used for feature selection are documented within the method section.
b) The images were contoured and analysed without reference to the outcome data.

All patients which met the inclusion criteria were included. (**Materials and methods, Results**)

Only complete data sets were used in the analysis. (**Results**)

. a) Clinical factors were included in the variable selection process alongside radiomic features, in each of the three predictive modelling techniques used. (**Statistical Analysis and Predictive Modelling**)

b) Each of the predictive modelling techniques used are detailed in the methods section. Univariate and forward wrapper feature selection methods were employed, as well as the exploration of principal components. (**Statistical Analysis and Predictive Modelling**)
d) Model performance was assessed using the C-index (**Statistical Analysis and Predictive Modelling**)

. Three risk groups were created within the model: high, medium and low risk groups. The survival probabilities, as well as the confidence intervals for each model are presented in the results. (**Results**)

. a) 187 patients from LTHT and 257 patients from CNFT were included, with demographics detailed in **Table 3.** (**Results**)
b) The characteristics of the participants are presented in **Table 3**.

. a) The number of events per cohort are presented in **Table 3**.
b) This has not been performed.

. a/b) The features and hyperparameters used to create the models are presented in the results section (**Results**).

. The C-indices, confidence intervals and survival probabilities for the different risk groups created from the predicted models created are presented. (**Results**)

. The limitations of the study are presented. These include the retrospective nature of the study, the relative low number of events, reliance on clinical records and the variation in radiomic extraction technique. (**Discussion**)

. b)/20. The discussion section gives an overall interpretation of the results and highlights the potential use of a pre-treatment model to aid in early personalised treatment for patients. (**Discussion**)

. The python libraries used are references within the text.

. The study was not externally funded. Individual author’s funding is declared within the **Declaration**.

Supplementary Material 2

Imaging acquisition and reconstruction

Studies performed at LTHT were acquired using standardised scanning parameters, as previously described [10]. All studies were performed using a standard imaging protocol for FDG PET-CT with acquisition from the skull base to the thighs. The unenhanced CT component was acquired with the following settings: 140 kV; 80 mAs; tube rotation time 0.5 s per rotation; pitch 6; 3.75mm section thickness. Patients were asked to maintain a normal breathing pattern throughout the study. Patients fasted for 6 hours prior to the study and glucose was checked prior to FDG injection. If the blood glucose was >10mmol/L the study was not performed, and the cause of raised glucose was addressed. Four different PET-CT scanners were used during the study period, detailed in **Supplementary Table 1**, along with the reconstruction parameters.

Studies performed at CNFT were performed using a standard FDG PET-CT imaging protocol with acquisition from the skull base to the thighs. Three different PET-CT scanners were used during the study period, with scanning and reconstruction parameters detailed in **Supplementary Table 1**. The CT component for the GE Discovery STE and the GE Discovery 710 were acquired with the following settings 120 kV; 15-220 mAs; tube rotation time 0.5 s per rotation; pitch 1.375-1.675; 3.75mm section thickness. The CT component for the Siemens Biograph TruePoint TrueV was acquired using the following settings: 130 kV; 45 mAs; tube rotation time 0.6 s per rotation; pitch 1.5; 4.0mm section thickness. Patients were asked to maintain a normal breathing pattern throughout the study. Patients fasted for 6 hours prior to the study and glucose was checked prior to FDG injection. If the blood glucose was >10mmol/L the study was abandoned and cause for the raised glucose addressed**.**

Image segmentation

At LTHT the segmentation of the images was performed by a single observer (clinical radiologist, 5 years' experience) under supervision of a dual-certified radiology and nuclear medicine physician (15 years' experience of oncological PET-CT). At CNFT the segmentation was performed by a single observer (clinical radiologist with 5 years' experience reporting PET-CT) following training and supervision from an oncological PET-CT reporter with 10 years' experience.

Scanner	Reconstruction	Scatter correction	Randoms correction	Matrix	Voxel size (x,y,z)
LTHT					
GE Healthcare STE	OSEM	Convolution subtraction	Singles	128	4.6875 x 4.6875 x 3.27
Philips Gemini TF64	BLOB-OS-TF	SS-Simul	DLYD	144 or 169	4 x 4 x 4
GE Healthcare Discovery 690	VPFX	Model based	Singles	192	3.65 x 3.65 x 3.27
GE Healthcare Discovery 710	VPFX	Model based	Singles	192	3.65 x 3.65 x 3.27
CNFT					
GE Healthcare Discovery STE	Vue Point HD	Convolution subtraction	Singles	128	5.47 x 5.47 x 3.75
Siemens Biograph TruePoint TrueV	Iterative	TrueC	Delayed	128	5.35 x 5.35 x 4.0
GE Healthcare Discovery 710	VPFX	Model based	Singles	192	3.65 x 3.65 x 3.27

Supplementary Table 1: Reconstruction parameters for the different scanners used at LTHT and CNFT. DLYD = delayed event subtraction, OSEM = ordered subsets expectation maximization, VPFX = Vue Point FX (3D Time of Flight), BLOB-OS-TF = 3D ordered subset iterative TOF reconstruction technique. SS-Simul is the CT based attenuation correction and scatter correction [9].

Shape	First Order	GLCM	GLRLM	GLSZM	NGTDM
Volume (mL)	Min	Homogeneity	Short run emphasises	Short-Zone Emphasis	Coarseness
Sphericity (only for 3D ROI (nZ>1)	Mean	Energy	Long run emphasises	Long-Zone Emphasis	Contrast
Compacity only for 3D ROI (nZ>1)	Std	Contrast	Low Grey-level Run Emphasis	Low Grey-level Zone Emphasis	Busyness
	Max	Correlation	High Grey-level Run Emphasis	High Grey-level Zone Emphasis	
	Skewness	Entropy (log10)	Short-Run Low Grey-level Emphasis	Short-Zone Low Grey-level Emphasis	
	Kurtosis	Entropy (log2)	Short-Run High Grey-level Emphasis	Short-Zone High Grey-level Emphasis	
	Entropy (log10)	Dissimilarity	Long-Run Low Grey-level Emphasis	Long-Zone Low Grey-level Emphasis	
	Entropy (log2)		Long-Run High Grey-level Emphasis	Long-Zone High Grey-level Emphasis	
	Energy		Grey-Level Non-Uniformity	Grey-Level Non-Uniformity	
			Run Length Non-Uniformity	Zone Length Non-Uniformity	
			Run Percentage	Zone Percentage	

Supplementary Table 2: detailing the radiomic features extracted for both the PET and CT components. The equations for the features are available at https://www.lifexsoft.org/index.php/resources/texture/radiomic-features?filter_tag%20[0]= (Last accessed 21/09/2022). Key: GLCM = grey level co-occurrence matrix, GLRLM = grey level run length matrix, GLSZM = grey level size zone matrix, NGTDM = neighbouring grey tone difference matrix

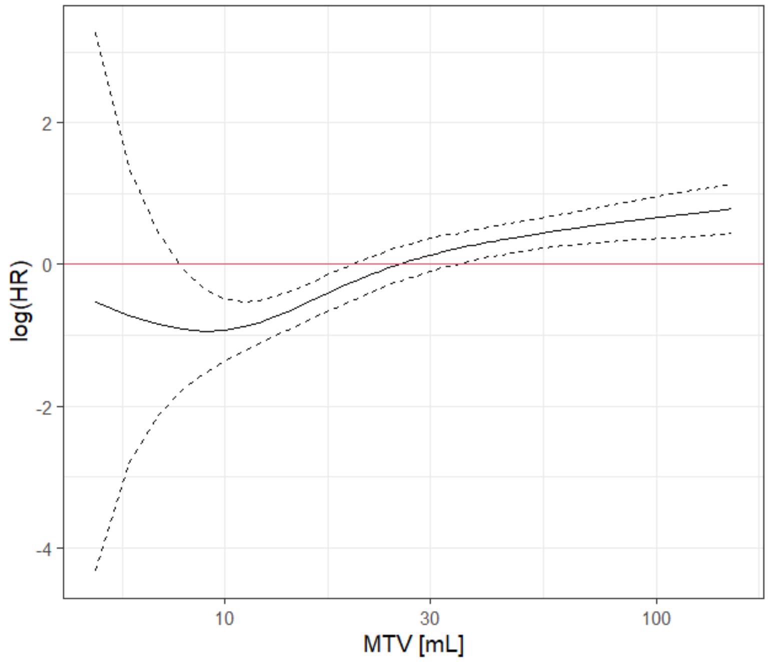

Supplementary Figure 1: Plot of the predictive log(hazard ratio(HR)) with increasing metabolic tumour volume (MTV) based on the LTHT dataset. The dashed lines represent the standard error.

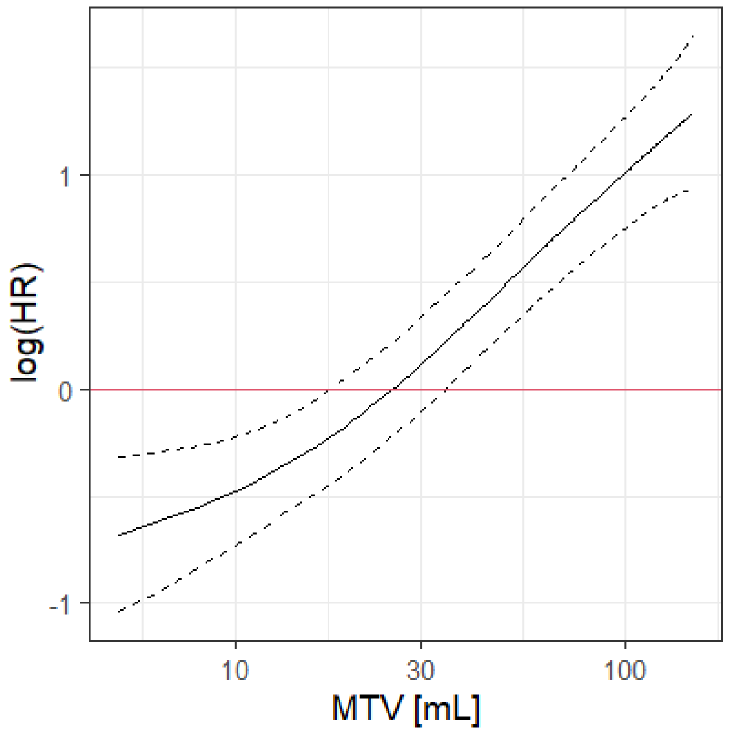

Supplementary Figure 2: Plot of the predictive log(hazard ratio(HR)) with increasing metabolic tumour volume (MTV) based on the CNFT dataset. The dashed lines represent the standard error.
